# Pravastatin-Induced Eczematous Eruption Mimicking Psoriasis

**DOI:** 10.1155/2017/3418204

**Published:** 2017-07-31

**Authors:** Michael P. Salna, Hannah M. Singer, Ali N. Dana

**Affiliations:** ^1^Department of Cardiothoracic Surgery, Stanford University School of Medicine, Stanford, CA, USA; ^2^Columbia University College of Physicians and Surgeons, New York City, NY, USA; ^3^Dermatology Service, James J. Peters VA Medical Center, Bronx, NY, USA; ^4^Department of Dermatology, Columbia University College of Physicians and Surgeons, New York City, NY, USA

## Abstract

**Background:**

Statins, an example of the most commonly prescribed medications to the elderly, are not without side effects. Dermatologic events are often overlooked as arising from medications, particularly those which are taken chronically. Moreover, elderly patients are prone to pharmacologic interactions due to multiple medications. In this report, we describe a case of a statin-induced eczematous dermatitis with a psoriasis-like clinical presentation and review the skin manifestations that may arise from statin therapy.

**Case Presentation:**

An 82-year-old man with gout and hypercholesterolemia presented to dermatology clinic with new onset of pruritic, scaly erythematous plaques bilaterally on the extensor surfaces of his arms. He had never had similar lesions before. Despite various topical and systemic treatments over several months, the rash continued to evolve. The patient was then advised to discontinue his long-term statin, which led to gradual resolution of his symptoms. He was subsequently diagnosed with statin-induced eczematous dermatitis.

**Conclusions:**

This case report describes an adverse cutaneous reaction to statins that is rarely reported in the literature. Medications, including longstanding therapies, should be suspected in cases of refractory dermatologic lesions.

## 1. Introduction

Statins have become the cornerstone of hypercholesterolemia pharmacotherapy in the elderly [1]. Despite the widespread use of these medications, their role in the development of new skin conditions may be overlooked and their dermatologic side effects misdiagnosed. Statins have been increasingly associated with drug-induced autoimmune reactions, including systemic lupus erythematosus, dermatomyositis, and lichen planus pemphigoides [2]. In this case report, we describe an unusual eczematous rash in an elderly patient on long-term pravastatin therapy that appeared several months after a dose increase.

## 2. Case Report

An 82-year-old man with a history of gout, hypercholesterolemia, and multiple nonmelanoma skin cancers was referred to our dermatology clinic for evaluation of pruritic lesions affecting the skin of the bilateral posterior arms for several weeks. He had no personal or family history of psoriasis or atopic dermatitis and denied any new contact exposures, new detergents, or starting any new medications or supplements. His daily medications included the following: allopurinol 300 mg, hydrochlorothiazide 25 mg, aspirin 81 mg, pravastatin 20 mg, and colchicine 0.6 mg. The patient had been on pravastatin therapy for hypercholesterolemia for over 5 years. Several months before onset of the rash his pravastatin had been increased from 10 mg to 20 mg daily.

**Figure 1:**
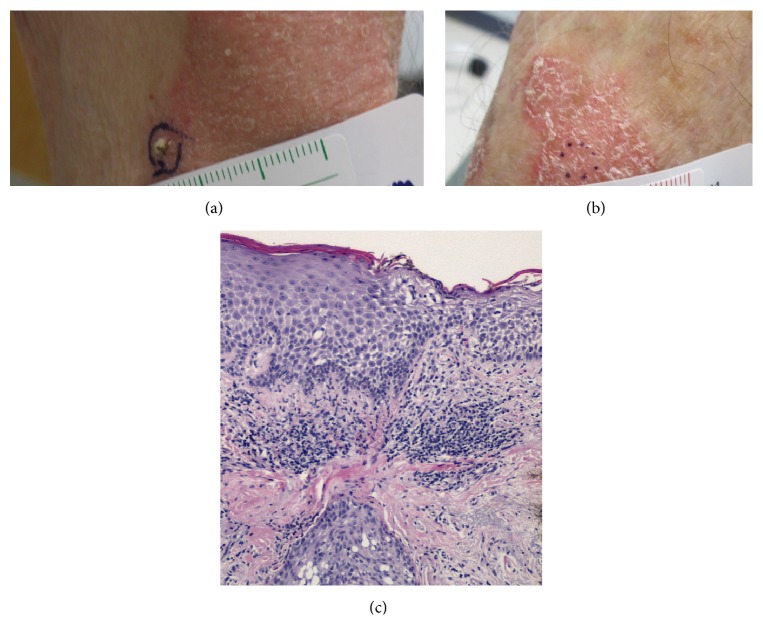
(a) Well-demarcated, erythematous patch with scale on the extensor surface of the right arm. (b) Dots indicate site of biopsy within lesion on the right arm. (c) Biopsy revealed spongiosis and perivascular lymphocytic infiltrate (hematoxylin and eosin).

Clinical examination revealed scaly, confluent, well-circumscribed erythematous plaques on the extensor surfaces of the upper arms extending to the forearms with no other lesions noted on skin exam (Figures 1(a) and 1(b)). Evaluation of the nails did not reveal any significant findings. A 4 mm punch biopsy was obtained from the forearm for routine histological evaluation. Given the psoriasis-like appearance, the patient was prescribed triamcinolone ointment 0.025%.

The biopsy (Figure 1(c)) revealed compact orthokeratosis and significant spongiosis with a perivascular lymphocytic infiltrate, consistent with an eczematous process. There was no evidence of acanthosis or psoriasiform epidermal hyperplasia. At the patient's follow-up appointment two weeks later, the lesions had spread to his anterior thighs and lower back. He was experiencing significant distress due to the pruritus and appearance of the rash and was treated with 40 mg intramuscular triamcinolone acetonide 0.1% solution. One month later, there was no improvement of his symptoms and alternative treatment options including phototherapy and nonsteroidal immunosuppressive therapy were considered. The patient was started on oral methotrexate, 10 mg weekly, as his schedule was unable to accommodate phototherapy. Further studies including complete blood count, metabolic panel, thyroid hormone, prostate specific antigen, serum protein electrophoresis, hepatitis B and hepatitis C, rapid plasma reagin, and autoimmune panel were unrevealing. Fungal culture and skin scraping for microscopic evaluation for dermatophyte were negative.

Despite an 8-week course of methotrexate, there was no improvement of his lesions. Finally, the patient underwent a trial off his pravastatin to rule out the statin as an etiologic agent. In collaboration with his primary care provider, the patient was switched to cholestyramine for cholesterol management and, after 8 weeks, his cutaneous lesions resolved. The patient was tapered off methotrexate and there was no recurrence of his lesions.

## 3. Discussion

Statins, an example of the most commonly prescribed medications in adults, lower lipid levels through inhibition of hepatic 3-hydroxy-3-methylglutaryl coenzyme-A reductase. Given the widespread usage of statins, diagnosticians should be aware of cutaneous reactions associated with this class of medications. The most commonly reported adverse dermatological reaction is a widespread, eczematous rash or hypersensitivity reaction. With respect to specific medications, atorvastatin has been reported to cause photosensitivity, edema, cheilitis, urticaria, and skin ulceration; lovastatin has caused pruritus and/or rash in up to 5% of patients; and simvastatin has been associated with lupus-like syndrome, lichenoid drug eruptions, cheilitis, photosensitivity, and vesiculobullous eruptions [3–5]. Any of the aforementioned statin-induced skin reactions can occur soon after initiation of therapy or after many years of stable dosing.

The mechanism of statin-induced cutaneous eruptions is not well understood. Statins have been noted to affect multiple immunological pathways, partially explaining the diversity of adverse cutaneous reactions that have been reported [4]. The anti-inflammatory effects of statins are mediated by inhibiting the expression of intercellular adhesion molecule-I on leukocytes and endothelial cells as well as suppressing antigen presentation and subsequent T-cell activation [6].

Drug interactions that affect the bioavailability and metabolism of statins may also account for variations in adverse cutaneous events. Simvastatin, lovastatin, atorvastatin, and rosuvastatin undergo metabolism in the liver by cytochrome P450 enzymes CYP3A4 and CYP2C9AV; however pravastatin does not undergo metabolism by CYP enzymes [6, 7]. Up to 60% of active pravastatin may undergo renal excretion due to its hydrophilic properties and relatively low binding fraction with plasma proteins. In serum, pravastatin has been found to have a lower rate of binding with plasma proteins of 43–48% compared to lovastatin, simvastatin, and fluvastatin which have plasma protein binding rates of at least 95%. Pravastatin has a lower fraction of hepatic extraction compared to simvastatin and lovastatin, which may result in increased exposure to pravastatin in peripheral tissues. Additionally, several pravastatin metabolites have activity comparable to the native drug. Of note, patients with hepatic failure have increased systemic exposure to all statins [8].

Systemic levels of pravastatin may also be affected by the activity of transport peptides. Permeability glycoprotein (P-gp) is an ATP-binding cassette transporter found in the small intestine, liver, kidneys, and blood-brain barrier. P-gp is located at luminal apical cells and acts on both endogenous organic compounds and many common medications, including pravastatin [9]. On enterocytes, apical P-gp is an efflux transporter that transports absorbed substrates back into the gut lumen for excretion. In hepatic biliary canaliculi, P-gp excretes substrates back into bile for elimination, and in renal tubules P-gp transports substances into urine [9, 10]. If the P-gp transporters become saturated with substrate then less medication will be eliminated and more will be absorbed systemically. Another transporter, the organic anion-transporting peptide 1B1 (OATP1B1), solute carriers also play a role in the movement of organic compounds and medications, including statins, into hepatocytes [7, 11]. OATP1B1 is located on both apical and basolateral ends of enterocytes and can translocate a variety of organic compounds and drugs. The specific role OATP1B1 and P-gp transporters play in absorption and metabolism of drug compounds is an area of active investigation with many clinical implications [10].

Case reports suggest that drug-drug interactions and genetic predispositions may precipitate adverse reactions for patients taking pravastatin; statins are very well tolerated overall. From searching the literature, three cases identified pravastatin as the cause of an adverse reaction [12, 15, 18]. All of the patients described were taking other medications, which may have influenced systemic exposure to pravastatin. Pravastatin is a substrate for the P-gp efflux transporter, and we outline in Table 1 the medications that are also reported to interface with P-gp. Drugs that are P-gp substrates, if saturated, would allow for more than expected absorption, as would inhibitors. P-gp inducers would decrease systemic exposure by increasing elimination.

## 4. Conclusion

This case demonstrates that pravastatin may be associated with psoriasis-like eczematous lesions that are resistant to treatment with steroids and immunosuppressive therapy. In any elderly patient with a new rash, it is worth remembering that both recently introduced and longstanding medications may play a role in skin changes and that the resolution of these changes may take weeks to months once the offending agent has been discontinued. Individual factors, slight differences between the metabolism and activity of statins, and interactions with other medication may all play a role in the etiology of statin-induced dermatitis.

## Figures and Tables

**Table 1:** Cases of pravastatin-induced adverse effects and P-glycoprotein interactions.

Reported case of pravastatin-induced adverse effect	Other medications taken by patient	P-glycoprotein effect	References
Lichenoid drug eruption in 64-year-old woman [12]	Aspirin	Possible inducer	[13]
	Candesartan	—	
	Clopidogrel	Substrate	[9]
	Diltiazem	Substrate, inhibitor, inducer	[9, 14]
	Furosemide	—	
	Hydralazine	—	
	Isosorbide mononitrate	—	
	Insulin	Inducer	[14]
	Metformin	—	

Diffuse, pruritic rash in a 55-year-old woman [15]	Aripiprazole	Substrate	[16]
	Lorazepam	—	
	Sertraline	Substrate and inhibitor	[9, 14, 16]
	Olanzapine	Substrate	[16]
	Quetiapine	Substrate	[16]
	Lithium carbonate	—	
	Levothyroxine	Inducer	[17]
	Magnesium	—	

Acute myopathy in a 65-year-old woman [18]	Aspirin	Possible inducer	[13]
	Colchicine	Substrate, inducer	[14, 18]
	Spironolactone	Inhibitor	[19]
	Furosemide	—	
	Losartan	Substrate, inhibitor	[9, 14]

Pruritic, psoriasis-like eczematous eruption in an 82-year-old man (current case)	Allopurinol	—	
	Aspirin	Possible inducer	[13]
	Colchicine	Substrate, inducer	[14, 18]
	Hydrochlorothiazide	—	
